# Hybrid laparo-endoscopic techniques for challenging colorectal lesions: a systematic review

**DOI:** 10.1007/s00464-025-12243-w

**Published:** 2025-09-29

**Authors:** Giovanni Distefano, Carlo Alberto Ammirati, Michele Barbiero, Roberto Passera, Alberto Arezzo

**Affiliations:** 1 Department of Surgical Sciences, University of Turin, Città della Salute e della Scienza di Torino, Turin, Italy; 2 Department of Medical Sciences, University of Turin, Turin, Italy; 3 Doctoral School of Bioengineering and Medico-Surgical Sciences, Politecnico di Torino, Turin, Italy

**Keywords:** Colorectal polyps, Laparo-endoscopic cooperative surgery, Hybrid resection, Minimally invasive surgery, Organ-preserving surgery

## Abstract

**Background:**

Colorectal cancer screening has increased the detection of polyps requiring resection, but standard endoscopic techniques such as endoscopic mucosal resection (EMR) or endoscopic submucosal dissection (ESD) are often unsuitable for large, fibrotic, or anatomically challenging lesions. Segmental colectomy remains definitive but carries substantial morbidity, particularly for benign disease. Laparo-endoscopic cooperative surgery (LECS) and related hybrid techniques have emerged as minimally invasive alternatives bridging the gap between endoscopic and surgical resection.

**Methods:**

A systematic review was performed according to PRISMA guidelines, querying PubMed, Embase, and Cochrane databases (1985–2024). Studies reporting combined laparoscopic-endoscopic resections for colorectal lesions unsuitable for standard endoscopic treatment were included. Outcomes assessed included additional surgery, adenocarcinoma detection, complication rates, surgery for complications, conversion to open surgery, and recurrence. Random-effects models were used to calculate pooled proportions and 95% confidence intervals (CIs).

**Results:**

Twenty-seven studies encompassing 1112 patients were included. The pooled rate of additional surgery was 5% (95% CI 3–8%; *I*^2^ = 0%), including 7% (95% CI 5–9%) for oncologic indications. Adenocarcinoma was identified in 12% of resected lesions (95% CI 8–16%), underscoring limitations of preoperative staging. Overall complications occurred in 7% (95% CI 5–10%), with surgery for complications required in only 1% (95% CI 0–2%). Conversion to open surgery occurred in 2% (95% CI 1–3%). Long-term follow-up demonstrated a local recurrence rate of 3% (95% CI 2–6%; *I*^2^ = 0%).

**Conclusions:**

Hybrid laparoscopic–endoscopic resections are safe, effective, and reproducible options for complex colorectal lesions not amenable to standard endoscopic resection. These techniques achieve low complication and recurrence rates while preserving bowel and minimizing morbidity associated with colectomy. Given the 12% incidence of unexpected adenocarcinoma, intraoperative adaptability and multidisciplinary expertise are essential. Prospective multicenter studies with standardized reporting are needed to refine patient selection and confirm long-term oncologic safety.

**Graphical abstract:**

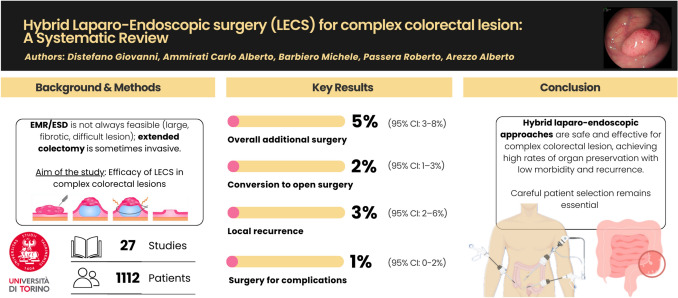

**Supplementary Information:**

The online version contains supplementary material available at 10.1007/s00464-025-12243-w.

Colorectal cancer (CRC) is the third most diagnosed malignancy and the second leading cause of cancer-related death globally. Worldwide, over 1.920.000 new CRC cases were reported in 2022 with approximately 904.000 deaths attributed to the disease [1]. Despite an overall decline in both incidence and mortality due to increased screening and improved management, regional disparities persist, largely reflecting unequal implementation of screening programs and differences in risk factor profiles [2].

The adenoma–carcinoma sequence remains the fundamental model for CRC development, with the majority of cancers arising from pre-existing adenomatous polyps or sessile serrated lesions [3]. Accordingly, early detection and endoscopic removal of these lesions represents the most effective strategy to reduce CRC incidence and mortality [4]. Large-scale colonoscopy screening programs have significantly increased the detection rate of colorectal polyps, leading to widespread adoption of endoscopic resection techniques such as polypectomy, endoscopic mucosal resection (EMR), and endoscopic submucosal dissection (ESD). These techniques are generally safe and effective; however, they may not be feasible for all lesions.

Large-scale colonoscopy screening programs have significantly increased the detection rate of colorectal polyps, leading to widespread adoption of endoscopic resection techniques such as endoscopic mucosal resection (EMR), and endoscopic submucosal dissection (ESD). These techniques are generally safe and effective; however, they may not be feasible for all lesions.

Large or “complex” polyps—typically defined as lesions > 20 mm, involving multiple folds or more than one-third of the colonic circumference, or located in anatomically challenging sites such as the right colon or near the ileocecal valve—are often associated with fibrosis due to prior resection attempts or are morphologically flat (e.g., lateral spreading tumors, LSTs). Such features substantially increase the technical difficulty and risk of complications during standard endoscopic resection. Additionally, piece-meal resection of large lesions often results in uncertain histopathological assessment and may compromise oncologic adequacy.

Although traditional surgical resection remains a definitive option for such difficult lesions, it is associated with increased morbidity and mortality, particularly in elderly patients or those with significant comorbidities. Segmental colectomy, while radical, is often unwarranted in cases of benign pathology. To bridge this gap, combined laparo-endoscopic approaches have been developed with the aim of minimizing surgical invasiveness while ensuring complete and safe resection of difficult colorectal lesions. Laparo-Endoscopic Cooperative Surgery (LECS) and similar collaborative procedures, including laparoscopically assisted endoscopic resection and endoscopic-assisted laparoscopic wedge or full-thickness resections, have gained attention in recent years. These techniques allow for intraoperative endoscopic localization and assessment of the lesion, laparoscopic control of the bowel wall, and selective full-thickness or transmural resection when needed. They offer the potential to preserve more bowel, avoid major colectomies, and enable immediate surgical management in the case of unexpected malignancy or perforation [5, 6].

Several retrospective and prospective series, as well as systematic reviews, have shown promising results in terms of efficacy, complication rates, and oncologic adequacy [7]. This evolving body of evidence highlights the potential role of laparo-endoscopic approaches in the management of complex colorectal neoplasia. However, considerable heterogeneity remains in terms of technique, indications, and reported outcomes. The present systematic review aims to assess the updated safety and efficacy profile of laparo-endoscopic resections for colorectal lesions that are considered unsuitable for standard endoscopic treatment, with a particular focus on recurrence, complications, oncologic outcomes, and the need for further interventions.

## Material and methods

This systematic review was conducted according to the Preferred Reporting Items for Systematic Reviews and Meta-Analyses (PRISMA) guidelines [8].

### Eligibility criteria

All studies published between January 1985 and December 2024 were eligible for inclusion if they reported on patients undergoing combined laparoscopic-endoscopic local resection of colorectal lesions. Eligible procedures included laparoscopically assisted endoscopic polypectomy, endoscopic-assisted laparoscopic wedge or full-thickness resection, and other minimally invasive techniques aimed at removing colorectal lesions unsuitable for standard endoscopic treatment.

Studies were excluded if they involved extended colorectal resections (e.g., segmental colectomies or hemicolectomies), included fewer than 10 patients, were animal studies, reviews, editorials, position papers, conference abstracts, or book chapters. In cases of suspected patient cohort overlap, only the most recent or complete publication was included.

### Search strategy

A comprehensive literature search was conducted in May 2025 using Embase, PubMed, and the Cochrane Central Register of Controlled Trials. The search string was specifically developed for this review and included terms related to laparoscopy, endoscopy, polypectomy, and colorectal lesions. The complete search strategy is provided as an appendix to this manuscript (Appendix 1).

### Study selection and data extraction

Two reviewers (GD, MB) independently screened all studies in a three-step process: first by title, then by abstract, and finally through full-text review of potentially eligible articles. Screening and selection were conducted using the Rayyan platform. Any discrepancies were resolved by consensus with a third reviewer (CAA).

Data extraction was independently performed by the same two reviewers using pre-defined data collection forms. Extracted variables included study design, country, sample size, patient demographics, lesion characteristics, surgical technique, recurrence, complications, conversion to open surgery, adenocarcinoma detection, and need for additional surgery (either for complications or oncologic reasons).

### Outcomes

The *primary outcome* of interest was the rate of additional surgery following combined laparoscopic–endoscopic resection. This included both reoperations for postoperative complications and surgeries performed to achieve oncologic radicality.

*Secondary outcomes* included:

- Incidence of adenocarcinoma in the resected specimens
- Rate of additional oncologic surgery
- Overall complication rate
- Rate of surgery due to complications
- Conversion rate to open surgery
- Recurrence rate after local resection

Each outcome was calculated as the proportion of lesions or patients meeting the criteria relative to the total included in the corresponding category.

### Quality assessment and statistical analysis

Risk of bias within individual studies was assessed using the Quality Assessment of Diagnostic Accuracy Studies (QUADAS) tool. Specifically, we evaluated the availability of histological reference standards, completeness of data regarding technical success, and reporting on oncologic adequacy.

Following a conservative approach, all outcomes were analysed by a random-effects model, where the proportions of single studies were used to calculate an overall proportion. This model incorporates heterogeneity among studies and takes into account differences in sample size by which proportions were measured in each study; this within-study variation was accounted for using the exact binomial distribution. Individual and pooled estimates of these proportions together with 95% confidence intervals (CIs) were presented in the Forest plots.

Publication bias was assessed by generating a funnel plot and performing the rank correlation test of funnel plot asymmetry. Heterogeneity was assessed by the *I*^2^ measure of inconsistency, statistically significant if *I*^2^ > 50%. Potential sources of heterogeneity were explored by two sensitivity analyses: checking the results of cumulative (sequentially including studies by date of publication) and influence analyses (calculating pooled estimates by omitting one study at a time). All analyses were performed using R 4.5.1 package meta (R Foundation for Statistical Computing, Vienna, Austria).

## Results

**Fig. 1:**
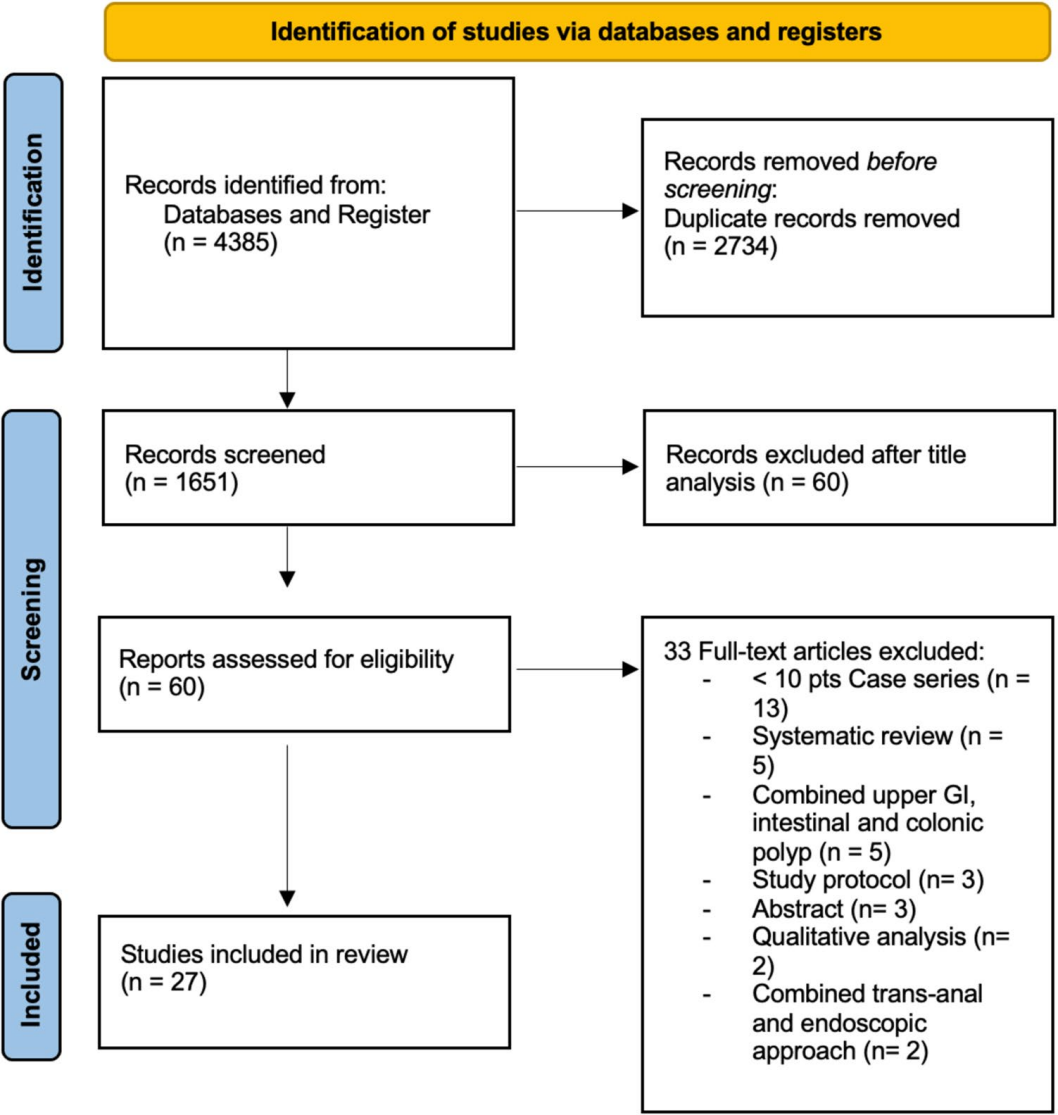
Flow-chart of the literature search in this systematic review

A total of 27 studies encompassing 1112 patients were included. A flow diagram of this systematic review, with the number of papers retrieved, included and excluded, as well as the reasons for exclusion, is shown in Fig. 1.

A developed summary table (Table 1) includes the core characteristics of all studies included in this review. The characteristics of the studies and detailed QUADAS quality assessment are also summarized in Table 1 and Supplementary Table 1, respectively. QUADAS evaluation showed generally high methodological quality: 100% of studies met core criteria such as appropriate patient spectrum, adequate reference standard, short test-reference interval, and blinded interpretation of results. Some variability was observed in reporting of selection criteria and procedural details (items 2, 8, 9), with 8 studies showing partial or unclear reporting.

**Table 1:** Core characteristics of all included studies

Reference	Study design	Country	Inclusion period	Mono/multicentric	Patients, n	Mean age (years)	Male (%)
Le Picard (1997) [9]	Prospective	France	1994–1996	Monocentric	16	56–80	56
Mal (1998) [10]	Retrospective	France	1990–1997	Monocentric	65	62	58
Ommer (2003) [11]	Retrospective	Germany	1995–2002	Monocentric	23	70.7	47.8
H. Winter (2007) [12]	Prospective	Germany	1998–2007	Monocentric	38	66	55
Wilhelm (2009) [13]	Prospective	Germany	1997–2006	Monocentric	146	64	47
Franklin (2009) [14]	Prospective	USA	1990–2008	Monocentric	160	74.7	51
DJ. Grunhagen (2011) [15]	Prospective	The Netherlands	2006–2009	Monocentric	11	73.2	63
Wood (2011) [16]	Prospective	UK	2008–2009	Monocentric	13	66	33
Yan (2011) [17]	Retrospective	USA	2003–2008	Monocentric	23	70	61
Jang (2012) [5]	Retrospective	USA	/	Monocentric	26	60.7	39
Sang W. Lee (2014) [18]	Retrospective	USA	2003–2012	Monocentric	75	69	43
C. Goh (2014) [19]	Retrospective	Ireland	2010–2013	Monocentric	30	65.4	60
Crawford (2015) [20]	Retrospective	Canada	2009–2013	Monocentric	30	64	66
Lascarides (2016) [21]	Prospective	USA	/	Monocentric	17	63	52
Račkauskas (2017) [22]	Retrospective	Lithuania	2010–2016	Monocentric	21	65.33	33
Tamegai (2018) [23]	Retrospective	Japan	2012–2018	Monocentric	17	66.5	58
Bulut (2019) [24]	Retrospective	New Zealand	2016–2017	Monocentric	25	71	52
S Suzuki (2019) [25]	Retrospective	Singapore	2004–2017	Monocentric	15	64	66
ES Huang (2020) [26]	Retrospective	USA	2013–2017	Monocentric	9	59.7	66.6
Parker (2021) [27]	Retrospective	UK	2008–2018	Monocentric	55	65	69
Kasim L Mirza (2021) [28]	Retrospective	California	2015–2020	Monocentric	22	64	81.8
Kolosov (2022) [29]	Prospective	Russia	2019–2020	Monocentric	31	67.8	93
Golda (2022) [30]	Retrospective	Spain	2010–2020	Monocentric	23	69.9	82.6
Serra-Aracil (2022) [31]	Retrospective	Spain	2018–2020	Monocentric	17	69	58
L W Leicher (2022) [32]	Prospective	The Netherland	2017–2019	Multicentric	110	66	56
Austin T Jones (2021) [33]	Retrospective	USA	2018–2019	Monocentric	37	67	59.5
Julia Hanevelt (2023) [34]	Retrospective	The Netherlands	2015–2022	Monocentric	57	69.5	61.4

Reporting of outcomes by specific hybrid technique was inconsistent across studies. For example, *Serra-Aracil *et al. [31] described different variants of combined endoscopic-laparoscopic surgery, including full-thickness resections and laparoscopically assisted resections, but did not provide stratified complication or recurrence rates. Similarly, *Suzuki *et al. [25] and *Tamegai *et al. [23] reported on LECS-CR procedures but presented results in aggregated form without separation by technique. This variability precluded meaningful subgroup analysis and limited the possibility of comparing outcomes across different hybrid approaches. For conceptual clarity, however, the hybrid procedures can be broadly categorized into three groups: (a) laparoscopically assisted EMR, (b) endoscopic-assisted laparoscopic wedge/full-thickness resections, and (c) endoscopic-assisted segmental resections.

### Primary outcome—additional surgery

**Fig. 2:**
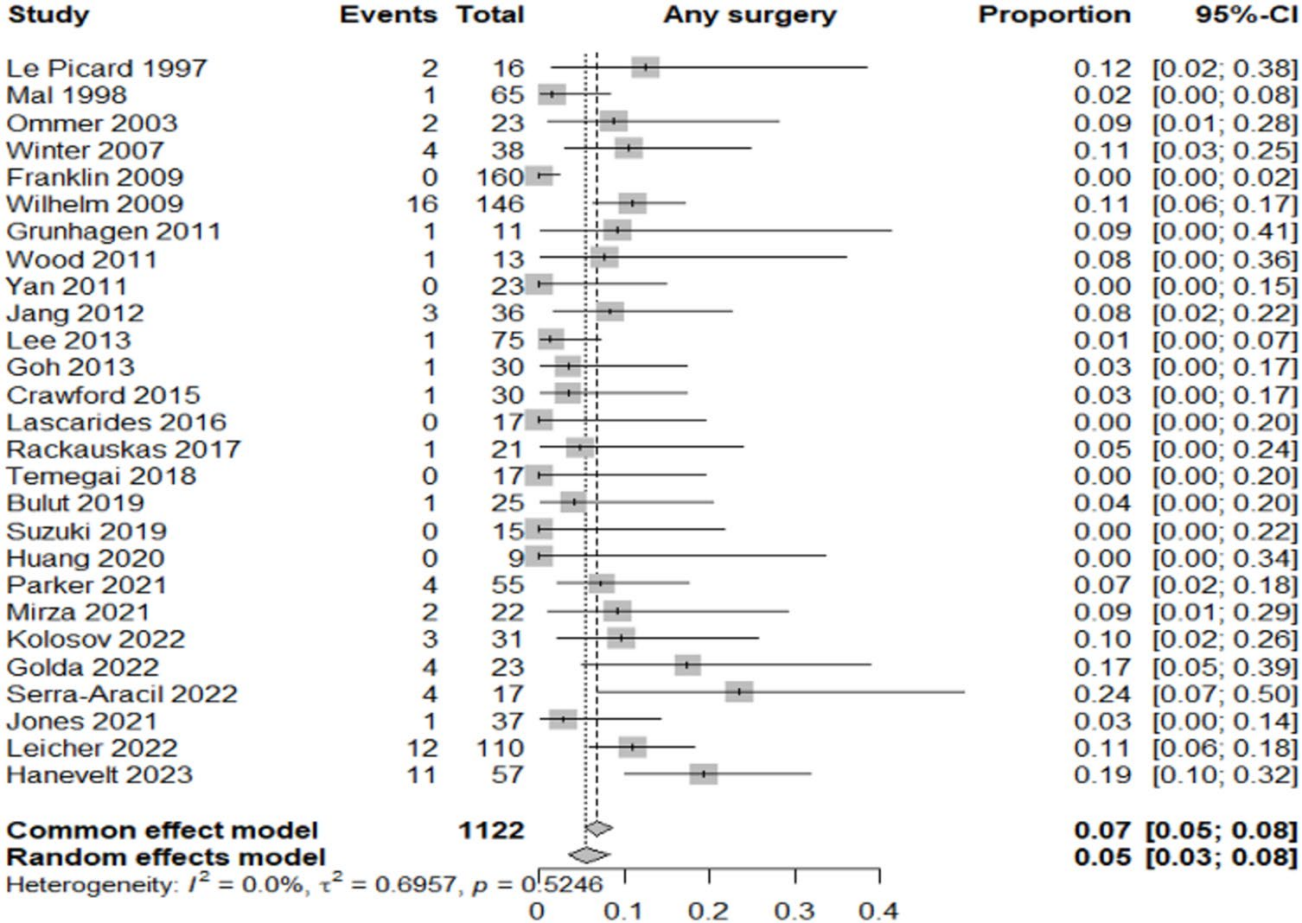
Forest plot for additional surgery

The overall rate of additional surgery, including both oncologic and complication-related interventions, was 5% (95% CI 3–8%; *I*^2^ = 0%), indicating excellent inter-study consistency. This represents a substantial reduction compared to standard segmental resections and supports the feasibility of the combined approach (Fig. 2).

### Secondary outcomes

Table 2 summarizes the proportion of lesions or patients meeting each outcome relative to the total included in the corresponding category.

**Fig. 3:**
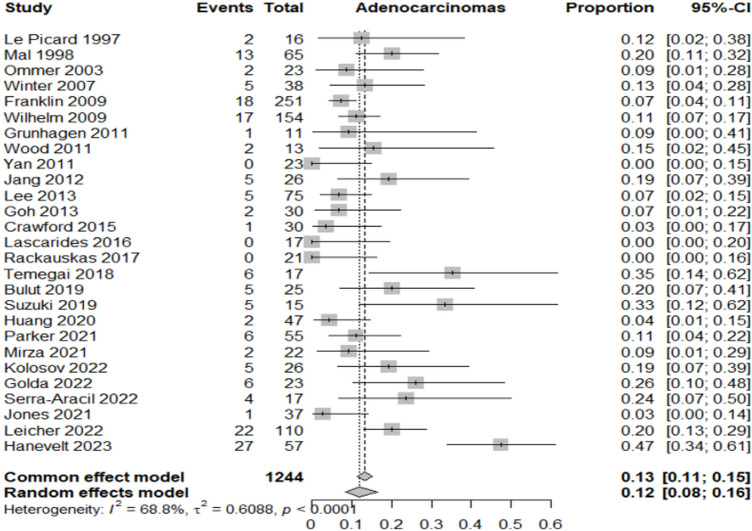
Forest plot for adenocarcinoma detection rate

**Fig. 4:**
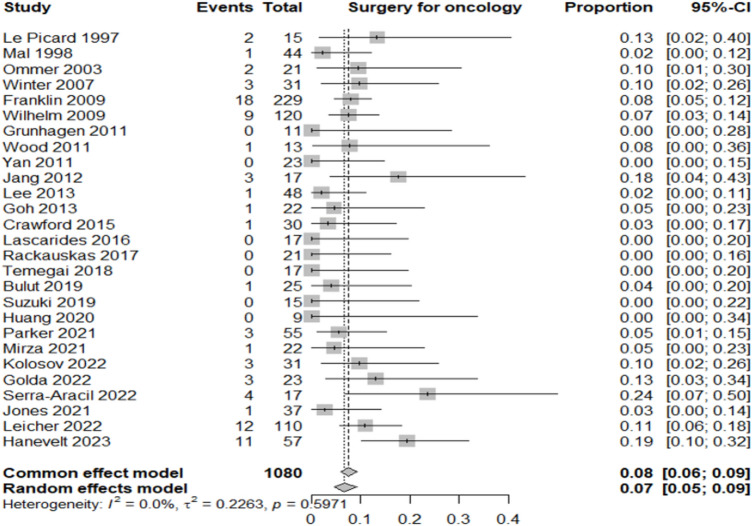
Forest plot for surgery for oncological reason

**Fig. 5:**
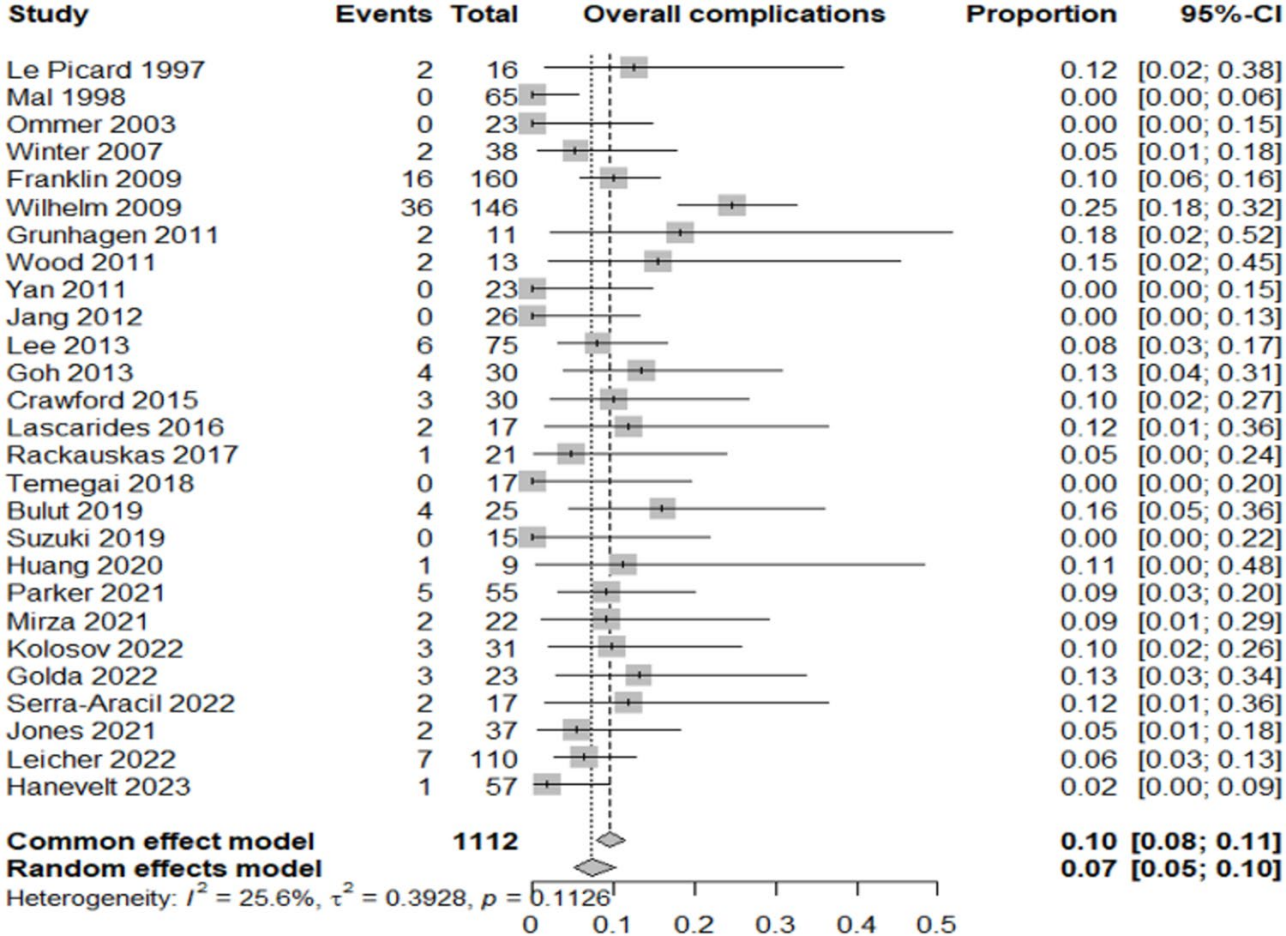
Forest plot for overall complications rate

**Fig. 6:**
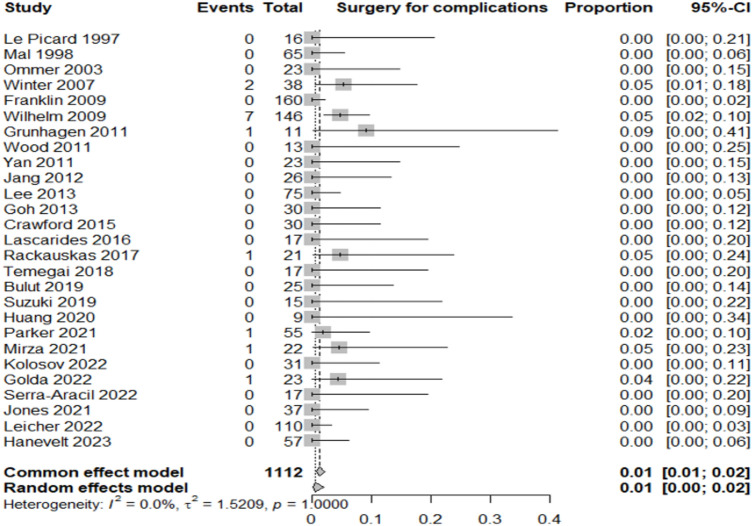
Forest plot for surgery for complications

**Fig. 7:**
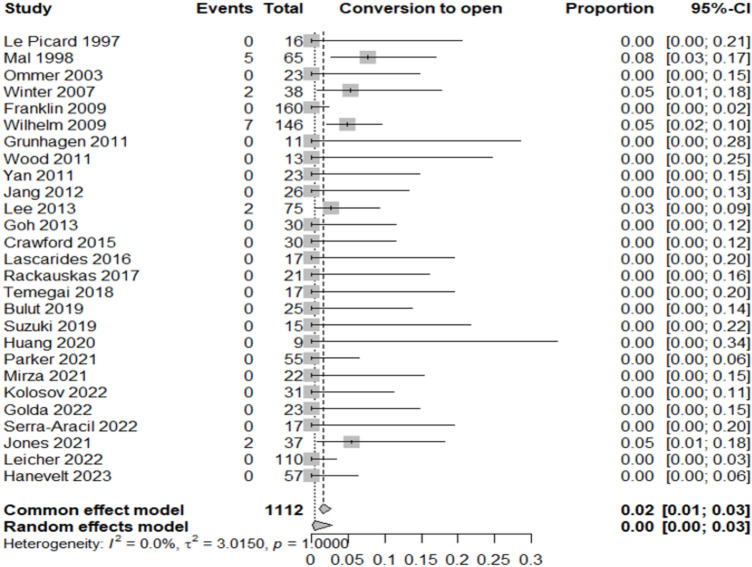
Forest plot for conversion to open surgery

**Fig. 8:**
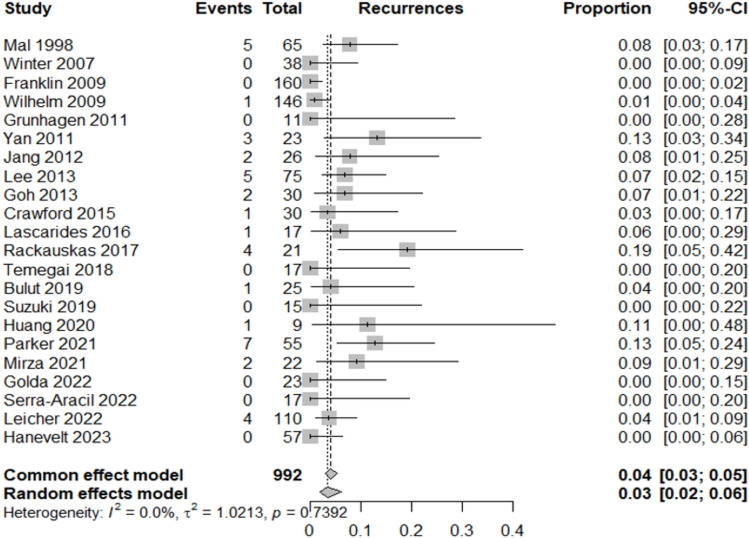
Forest plot for local recurrence rate

- Adenocarcinoma detection rate: adenocarcinoma was identified in 12% of resected lesions (95% CI 8–16%; *I*^2^ = 68.8%), highlighting significant heterogeneity in preoperative diagnostic accuracy across studies (Fig. 3).
- Oncologic surgery requirement: following local resection, 7% of patients (95% CI 5–9%; *I*^2^ = 0%) required radical oncologic surgery, primarily due to invasive histology (Fig. 4).
- Overall complication rate: complications occurred in 7% of patients (95% CI 5–10%; *I*^2^ = 25.6%), mainly bleeding, perforation, and localized infections (Fig. 5).
- Surgery for complications: surgical management was necessary in 1% of cases (95% CI 0–2%; *I*^2^ = 0%) (Fig. 6).
- Conversion to open surgery: conversion was required in 2% of procedures (95% CI 1–3%; *I*^2^ = 0%), confirming good intraoperative control even in technically challenging situations (Fig. 7).
- Local recurrence: long-term follow-up of 992 patients showed a local recurrence rate of 3% (95% CI 2–6%; *I*^2^ = 0%), indicating durable local control (Fig. 8).

**Table 2:** Main results of the analyzed outcomes

Analyzed variables	Value (%)	95% CI	*I*^2^ (%)
Recurrence rate	3	2–6	0
Adenocarcinoma detection rate	12	8–16	68.8
Overall complications rate	7	5–10	25.6
Surgery for complications	1	0–2	0
Conversion to open surgery	2	1–3	0
Oncologic surgery requirement	7	5–9	0

Follow-up duration was reported in 18 out of 27 studies, with a median of 20 months (range 1–196 months). Although reporting methods varied across studies, a sensitivity analysis restricted to studies with at least 12 months of follow-up yielded recurrence rates comparable to the overall analysis, thereby confirming the robustness of our results.

## Discussion

This systematic review confirms that combined laparoscopic-endoscopic techniques, such as LECS and its variants, are safe and effective for the treatment of complex colorectal lesions that are not suitable for standard endoscopic resection. Hybrid laparo-endoscopic approaches encompass a wide spectrum of techniques, ranging from laparoscopically assisted EMR to wedge or full-thickness resections and more limited segmental resections. Although some recent reports, such as *Serra-Aracil* et al. [31], have highlighted these variants, outcome measures were usually presented in aggregated form without stratification by technique. This lack of granularity precluded reliable meta-analytic comparison between procedural subtypes, and our pooled estimates should therefore be interpreted as representative of hybrid approaches as a whole rather than of individual procedures. With a pooled additional surgery rate of just 5% and consistent results across studies, this approach appears to provide a reliable organ-preserving alternative to traditional segmental colectomy, particularly when complete endoscopic resection is either infeasible or carries high risk. Recent technological advancements and growing expertise in the field of endoscopic resection of large superficial colorectal lesions are progressively reducing the need for surgical intervention. According to recent data from high-volume European centers, endoscopic submucosal dissection (ESD) achieves en bloc resection rates exceeding 85% even for lesions larger than 20 mm, although the true oncologic benefit is realized in only a limited subset of patients.

Endoscopic Submucosal Dissection (ESD) has been widely adopted for en bloc resection of superficial colorectal neoplasms; however, its clinical benefit is often undermined by the relatively high incidence of incomplete (R1) resections. Reported R1 rates in colorectal ESD range from 8 to 15%, largely due to technical challenges, fibrosis, and lesion morphology in the colon and rectum, which differ substantially from gastric ESD where en bloc R0 rates exceed 90% [35–37]. Incomplete resection not only negates the oncologic advantage of ESD by leaving positive margins but also necessitates additional interventions, either repeat endoscopic procedures or radical surgery, thereby increasing morbidity [38]. Moreover, several studies have shown that R1 resections are associated with a significantly higher risk of local recurrence compared to R0 resections [39]. These limitations underscore the need for careful patient selection and technical refinement, as well as the potential role of hybrid laparoscopic-endoscopic approaches to ensure complete full-thickness resection in anatomically or technically challenging lesions [40]. Specifically, the primary oncologic advantage of ESD over endoscopic mucosal resection (EMR) is seen in cases of well-differentiated adenocarcinomas with superficial submucosal invasion and no lymphovascular involvement. However, due to its technical complexity and higher risk of adverse events, ESD should be reserved for lesions with a high suspicion of early submucosal invasion [41]. In most other cases, piecemeal EMR remains a safe and sufficient option for removing large (> 2 cm), sessile or flat colonic lesions. If histology reveals invasive adenocarcinoma beyond the mucosal layer, radical surgery is recommended. Conversely, the presence of dysplasia at the resection margin does not justify surgery but rather calls for close endoscopic follow-up.

The findings also highlight that a significant proportion of lesions presumed benign—approximately 12%—were found to harbor adenocarcinoma on final pathology. The heterogeneity observed in the pooled incidence of adenocarcinoma is mainly attributable to differences in diagnostic accuracy and patient selection across studies. In most series, adenocarcinoma was diagnosed only a posteriori on the final resection specimen, whereas preoperative biopsies rarely guided the indication for hybrid resection and were reserved for patients unfit for extended colectomy. As endoscopic biopsy usually samples only a fraction of the lesion, it may easily understage the true histology, explaining why invasive carcinoma was unexpectedly identified in lesions considered benign at endoscopy. For this reason, a reliable subgroup analysis stratifying lesions as benign versus malignant at baseline could not be performed, as such categories were not consistently available prior to resection. This diagnostic limitation must be acknowledged as a key contributor to the heterogeneity observed in the pooled analysis.

This underscores the limitations of current diagnostic modalities in precisely staging complex colorectal polyps, despite advances in imaging and biopsy techniques. Consequently, a second surgical procedure for oncologic radicality was necessary in 7% of cases. Intraoperative frozen section examination was not reported in any of the included studies. Consequently, adenocarcinoma was generally recognized only on final pathology of the hybrid resection specimen. Patients with such findings were then offered oncologic resection whenever clinically appropriate and acceptable. This highlights both the limits of current intraoperative decision-making and the potential for understaging with endoscopic biopsy, which samples only a small fraction of large or complex lesions. The absence of frozen section use in published series may have contributed to the proportion of patients requiring secondary oncologic surgery observed in this review. This outcome, while not negligible, remains acceptable in a clinical scenario where overtreatment through upfront colectomy can be avoided in the majority of patients. The long-term local recurrence rate of 3% (95% CI 2–6%; *I*^2^ = 0%) observed in this systematic review underscores the durability of local control achieved with these techniques. This exceptionally low rate, coupled with the absence of heterogeneity across included studies, highlights the oncologic reliability of these approaches when applied in appropriately selected patients. Such outcomes are comparable to, or even better than, those reported for standard surgical resections in early colorectal neoplasia, supporting their role as effective organ-preserving strategies. Moreover, the consistency of results across studies suggests that these techniques can deliver reproducible oncologic safety in different clinical settings, provided that meticulous patient selection and adherence to technical standards are ensured. Only relevant improvements in intraoperative staging of rectal cancer at the time of local excision, such as the use of fluorescence to better characterise tumour histology and invasiveness [42], or sampling of potential sentinel lymph nodes in the mesorectum [43–45], could significantly alter the perspective on organ-sparing techniques. Moreover, these techniques may take advantage of recent research in AI and computed tomography (CT) in assisting the preoperative localization of colorectal cancer resection surgery [46].

The complication rate observed in this review was low, with only 7% of patients experiencing adverse events and a mere 1% requiring surgical management of complications. These results compare favorably with those of standard surgical resection and suggest that laparo-endoscopic approaches maintain a favorable safety profile, even in anatomically challenging cases such as lesions located in the right colon, near the ileocecal valve, or involving previous fibrosis. Moreover, conversion to open surgery was required in only 2% of procedures, indicating that combined approaches are technically feasible and controllable when performed by adequately trained surgical and endoscopic teams. Perhaps most notably, the recurrence rate was just 3%, with excellent consistency across studies, suggesting durable local control and confirming the oncologic reliability of these techniques.

The methodological quality of the included studies was assessed using the QUADAS tool, which revealed an overall high level of internal validity. All studies adequately described the patient spectrum and ensured the independence and reliability of the reference standard. However, certain aspects, such as the clarity of selection criteria and the level of detail provided about index and reference test execution, were less consistently reported. These variations in reporting standards point to a broader need for methodological rigor and transparency in future research.

Despite these strengths, this review has several limitations that must be considered when interpreting the results. The majority of studies were retrospective and monocentric, often conducted in high-volume referral centers, which may limit generalizability to lower-volume or community settings. There was also considerable heterogeneity in the technical execution of the procedures classified under the laparo-endoscopic umbrella, ranging from endoscopic-assisted wedge resections to laparoscopically assisted EMR and full-thickness resections. Follow-up periods varied across studies, and long-term outcomes such as disease-free survival or metachronous lesion development were not systematically reported, making it difficult to draw firm conclusions about oncologic prognosis beyond recurrence. Only a few studies provided extended follow-up beyond 30 months [13, 14, 18, 23, 27], consistently reporting low local recurrence rates. Overall, the pooled recurrence rate across all studies was about 3%, suggesting durable local control and serving as a proxy for oncologic reliability. However, detailed long-term oncologic endpoints such as disease-free survival (DFS) and overall survival (OS) were rarely reported and inconsistently presented across studies. This gap limits the ability to establish oncologic equivalence with standard colectomy and underscores the need for prospective trials with standardized DFS and OS reporting. Furthermore, some studies lacked sufficient detail regarding inclusion criteria and procedural methodology, limiting the ability to stratify results by lesion type or location. Nevertheless, the consistency in core outcomes—such as low recurrence, minimal complications, and a stable rate of additional oncologic surgery—suggests that laparo-endoscopic approaches offer significant advantages when appropriately indicated and performed by experienced multidisciplinary teams.

Hybrid laparoscopic-endoscopic resection techniques emerge as a compelling option for the management of complex colorectal lesions that are not amenable to standard endoscopic removal. These hybrid approaches allow for effective and safe local treatment while minimizing the need for extensive bowel resection. The low rates of complications, recurrence, and conversion to open surgery observed across studies highlight their feasibility and reproducibility in expert settings. The not insignificant proportion of patients found to have invasive carcinoma underscores the need for accurate preoperative evaluation and appropriate intraoperative adaptability. Still, the overall outcomes suggest that the benefits of local, minimally invasive resection can be preserved even when a secondary oncologic surgery becomes necessary. Ultimately, laparo-endoscopic cooperative surgery should be considered an integral part of the therapeutic armamentarium for colorectal lesions, bridging the gap between pure endoscopy and radical surgery. Future prospective, multicenter studies with standardized definitions, longer follow-up, and consistent reporting are needed to refine indications and further establish the long-term oncologic safety of this evolving approach.

This systematic review provides robust evidence supporting the safety and efficacy of combined laparoscopic-endoscopic approaches, such as LECS and its variants, for the management of complex colorectal lesions not suitable for standard endoscopic resection. The findings align with recent trends in minimally invasive surgery, confirming the role of hybrid techniques as a bridge between endoscopic and formal surgical management.

## Supplementary Information

Below is the link to the electronic supplementary material.

Supplementary file1 (DOCX 28 KB)

Supplementary file2 (DOCX 22 KB)

## References

[1] Li JJ, Zhang YM, Ji YT, Wu J, Jin QY, Feng ZW, Duan HY, Liu XM, Lyu ZY, Song FJ, Huang YB (2025) Comparison analyses of global burden of colorectal cancer. Zhonghua Zhong Liu Za Zhi 47(4):308–315. 10.3760/cma.j.cn112152-20240308-00102. (**Chinese**) 40268547 10.3760/cma.j.cn112152-20240308-00102

[2] Zorzi M, Dal Maso L, Francisci S et al (2019) AIRTUM Working Group. Trends of colorectal cancer incidence and mortality rates from 2003 to 2014 in Italy. Tumori 105(5):417–426. 10.1177/0300891619838336 30917756 10.1177/0300891619838336

[3] Muto T, Bussey HJ, Morson BC (1975) The evolution of cancer of the colon and rectum. Cancer 36(6):2251–2270. 10.1002/cncr.2820360944 1203876 10.1002/cncr.2820360944

[4] Wagle NS, Nogueira L, Devasia TP, Mariotto AB, Yabroff KR, Islami F, Jemal A, Alteri R, Ganz PA, Siegel RL (2025) Cancer treatment and survivorship statistics, 2025. CA Cancer J Clin 75(4):308–340. 10.3322/caac.70011 40445120 10.3322/caac.70011 PMC12223361

[5] Jang JH, Kirchoff D, Holzman K, Park K, Grieco M, Cekic V, Naffouje S, Kluft J, Whelan RL (2012) Laparoscopic-facilitated endoscopic submucosal dissection, mucosal resection and partial circumferential (“Wedge”) colon wall resection for benign colorectal neoplasm that come to surgery. Surg Innov 20:234–240 22858573 10.1177/1553350612456098

[6] Nakajima K, Sharma SK, Lee SW, Milsom JW (2016) Avoiding colorectal resection for polyps: is CELS the best method? Surg Endosc 30:807–818 26092011 10.1007/s00464-015-4279-6

[7] Arezzo A, Passera R, Migliore M, Cirocchi R, Galloro G, Manta R, Morino M (2015) Efficacy and safety of laparo-endoscopic resections of colorectal neoplasia: a systematic review. United Eur Gastroenterol J 3(6):514–522. 10.1177/2050640615581967 10.1177/2050640615581967 PMC4669514 26668744

[8] Liberati A, Altman DG, Tetzlaff J, Mulrow C, Gøtzsche PC, Ioannidis JP, Clarke M, Devereaux PJ, Kleijnen J, Moher D (2009) The PRISMA statement for reporting systematic reviews and meta-analyses of studies that evaluate health care interventions: explanation and elaboration. Ann Intern Med 151:65–94 10.7326/0003-4819-151-4-200908180-00136 19622512

[9] Le Picard P, Vacher B, Pouliquen X (1997) Laparoscopy-assisted colonic polypectomy or how to be helped by laparoscopy to prevent colectomy in benign colonic polyps considered to be unresectable by colonoscopy. Ann Chir 51:986–989 10868040

[10] Mal F, Perniceni T, Levard H, Levy P, Gayet B (1998) Colonic polyps considered unresectable by endoscopy: removal by combinations of laparoscopy and endoscopy in 65 patients [in French]. Gastroenterol Clin Biol 22:425–430 9762273

[11] Ommer A, Limmer J, Mollenberg H, Peitgen K, Albrecht KH, Walz MK (2003) Laparoscopic-assisted colonoscopic polypectomy: indications and results. Zentralbl Chir 128:195–198 12695924 10.1055/s-2003-38531

[12] Winter H, Lang RA, Spelsberg FW, Jauch KW, Hüttl TP (2007) Laparoscopic colonoscopic rendezvous procedures for the treatment of polyps and early-stage carcinomas of the colon. Int J Colorectal Dis 22:1377–1381 17646999 10.1007/s00384-007-0345-4

[13] Wilhelm D, von Delius S, Weber L, Meining A, Schneider A, Friess H, Schmid RM, Frimberger E, Feussner H (2009) Combined laparoscopic-endoscopic resections of colorectal polyps: 10-year experience and follow-up. Surg Endosc 23:688–693 19169747 10.1007/s00464-008-0282-5

[14] Franklin ME Jr, Portillo G (2009) Laroscopic monitored colonoscopic polypectomy: long-term follow-up. World J Surg 33:1306–1309 19280252 10.1007/s00268-009-9967-8

[15] Grünhagen DJ, van Ierland MC, Doornebosch PG, Bruijninckx MM, Winograd R, de Graaf EJ (2011) Laparoscopic monitored colonoscopic polypectomy: a multimodality method to avoid segmental colon resection. Colorectal Dis 13(11):1280–1284 21091600 10.1111/j.1463-1318.2010.02515.x

[16] Wood JJ, Lord AC, Wheeler JMD, Borley NR (2011) Laparo-endoscopic resection for extensive and inaccessible colorectal polyps: a feasible and safe procedure. Ann R Coll Surg Engl 93:241–245 21477440 10.1308/003588411X565978 PMC3291144

[17] Yan J, Trecheva K, Lee SW, Sonoda T, Shukla P, Milsom JW (2011) Treatment for right colon polyps not removable using standard colonoscopy: combined laparoscopic-colonoscopic approach. Dis Colon Rectum 54:755–758 10.1007/DCR.0b013e3182108289 21552062

[18] Lee SW, Garrett KA, Shin JH, Trencheva K, Sonoda T, Milsom JW (2013) Dynamic article: long-term outcomes of patients undergoing combined endolaparoscopic surgery for benign colon polyps. Dis Colon Rectum 56(7):869–873 23739193 10.1097/DCR.0b013e3182821e58

[19] Goh C, Burke JP, McNamara DA, Cahill RA, Deasy J (2014) Endolaparoscopic removal of colonic polyps. Colorectal Dis 16(4):271–275 24308442 10.1111/codi.12512

[20] Crawford AB, Yang I, Wu RC, Moloo H, Boushey RP (2015) Dynamic article: combined endoscopic-laparoscopic surgery for complex colonic polyps: postoperative outcomes and video demonstration of 3 key operative techniques. Dis Colon Rectum 58(3):363–369 25664717 10.1097/DCR.0000000000000311

[21] Lascarides C, Buscaglia JM, Denoya PI, Nagula S, Bucobo JC, Bergamaschi R (2016) Laparoscopic right colectomy vs laparoscopic-assisted colonoscopic polypectomy for endoscopically unresectable polyps: a randomized controlled trial. Colorectal Dis 18(11):1050–1056 27038277 10.1111/codi.13346

[22] Račkauskas R, Mikalauskas S, Petrulionis M, Poškus T, Jotautas V, Stanaitis J, Poškus E, Strupas K (2017) Laparoscopically assisted colonoscopic polypectomy—viable option for curative surgery in elderly patients. Wideochir Inne Tech Maloinwazyjne 12(2):120–124 28694896 10.5114/wiitm.2017.68138 PMC5502343

[23] Tamegai Y, Fukunaga Y, Suzuki S, Lim DNF, Chino A, Saito S, Konishi T, Akiyoshi T, Ueno M, Hiki N, Muto T (2018) Laparoscopic and endoscopic cooperative surgery (LECS) to overcome the limitations of endoscopic resection for colorectal tumors. Endosc Int Open 6(12):E1477–E1485 30574538 10.1055/a-0761-9494 PMC6291397

[24] Bulut M, Knuhtsen S, Holm FS, Eriksen JR, Gögenur I, Bremholm L (2019) Combined endoscopic laparoscopic surgical treatment of advanced adenomas and early colon cancer. Dan Med J 66(8):A5562 31315798

[25] Suzuki S, Fukunaga Y, Tamegai Y, Akiyoshi T, Konishi T, Nagayama S, Saito S, Ueno M (2019) The short-term outcomes of laparoscopic-endoscopic cooperative surgery for colorectal tumors (LECS-CR) in cases involving endoscopically unresectable colorectal tumors. Surg Today 49(12):1051–1057 31250113 10.1007/s00595-019-01840-7

[26] Huang ES, Chumfong IT, Alkoraishi AS, Munroe CA (2020) Combined endoscopic mucosal resection and extended laparoscopic appendectomy for the treatment of periappendiceal, cecal, and appendiceal adenomas. J Surg Res 252:89–95 32278221 10.1016/j.jss.2020.02.006

[27] Parker J, Torkington J, Davies MM, Dolwani S (2021) Laparoscopically assisted endoscopic mucosal resection reduces the need for bowel resection for complex colonic polyps. Br J Surg 108(5):e196–e198 33638645 10.1093/bjs/znab053

[28] Mirza KL, Wickham CJ, Noren ER, Hin J, Cologne KG, Lee SW (2021) Full-thickness laparoendoscopic excision for management of complex colon polyps. Dis Colon Rectum 64(12):1559–1563 34596631 10.1097/DCR.0000000000002112

[29] Kosolov AV, Sushkov OI, Surovegin ES, Likutov AA, Kashnikov VN, Yugai OM, Achkasov SI (2022) Hybrid laparo-endoscopic surgery for colon tumors (results of pilot study). Koloproktologia 21(1):83–88

[30] Golda T, Lazzara C, Sorribas M, Soriano A, Frago R, Alrasheed A, Kreisler E, Biondo S (2022) Combined endoscopic-laparoscopic surgery (CELS) can avoid segmental colectomy in endoscopically unremovable colonic polyps: a cohort study over 10 years. Surg Endosc 36(1):196–205 33439344 10.1007/s00464-020-08255-3

[31] Serra-Aracil X, Gil-Barrionuevo E, Martinez E, Mora-López L, Pallisera-Lloveras A, Serra-Pla S, Puig-Divi V, Navarro-Soto S (2022) Combined endoscopic and laparoscopic surgery for the treatment of complex benign colonic polyps (CELS): observational study. Cir Esp (Engl Ed) 100(4):215–222 35431169 10.1016/j.cireng.2022.03.005

[32] Leicher LW, Huisman JF, van Grevenstein WMU, Didden P, Backes Y, Offerhaus GJA, Laclé MM, Moll FCP, Geesing JMJ, Smakman N, Droste JSTS, Verdaasdonk EGG, Ter Borg F, Talsma AK, Erkelens GW, van der Zaag ES, Schrauwen RW, van Wely BJ, Schot I, Vermaas M, van Bergeijk JD, Sietses C, Hazen WL, Wasowicz DK, Ramsoekh D, Tuynman JB, Alderlieste YA, Renger RJ, Oort FA, Bilgen EJS, Vleggaar FP, Vasen HFA, de Vos Tot Nederveen Cappel WH, Moons LMG, van Westreenen HL (2022) Colonoscopic-assisted laparoscopic wedge resection for colonic lesions: a prospective multicenter cohort study (LIMERIC-study). Ann Surg 275(5):933–939 35185125 10.1097/SLA.0000000000005417

[33] Jones AT, Broome JM, Zelhart MD (2022) Combined endoscopic robotic surgery for complex colonic polyp resection: case series. Surg Endosc 36(6):3852–3857 34494158 10.1007/s00464-021-08702-9

[34] Hanevelt J, Moons LMG, Hentzen JEKR, Wemeijer TM, Huisman JF, de Vos Tot Nerveen Cappel WH, van Westreenen HL (2023) Colonoscopy-assisted laparoscopic wedge resection for the treatment of suspected T1 colon cancer. Ann Surg Oncol 30(4):2058–2065 36598625 10.1245/s10434-022-12973-4

[35] Tanaka S, Terasaki M, Kanao H, Oka S, Chayama K (2012) Current status and future perspectives of endoscopic submucosal dissection for colorectal tumors. Dig Endosc 24(Suppl 1):73–79. 10.1111/j.1443-1661.2012.01252.x 22533757 10.1111/j.1443-1661.2012.01252.x

[36] Fuccio L, Hassan C, Ponchon T, Mandolesi D, Farioli A, Cucchetti A, Frazzoni L, Bhandari P, Bellisario C, Bazzoli F, Repici A (2017) Clinical outcomes after endoscopic submucosal dissection for colorectal neoplasia: a systematic review and meta-analysis. Gastrointest Endosc 86(1):74–86.e17. 10.1016/j.gie.2017.02.024 28254526 10.1016/j.gie.2017.02.024

[37] Chen Y, Wu Z (2025) The efficacy and safety of precutting-endoscopic mucosal resection for colorectal tumors: a systematic review and meta-analysis. Minim Invasive Ther Allied Technol 34(3):177–186. 10.1080/13645706.2024.2440403 39668459 10.1080/13645706.2024.2440403

[38] Oh HH, Kim JS, Lim JW, Lim CJ, Seo YE, You GR, Im CM, Kim KH, Kim DH, Kim HS, Joo YE (2024) Clinical outcomes of colorectal neoplasm with positive resection margin after endoscopic submucosal dissection. Sci Rep 14(1):12353. 10.1038/s41598-024-63129-1 38811758 10.1038/s41598-024-63129-1 PMC11136969

[39] Gupta S, Burgess NG (2025) Recurrence following piecemeal endoscopic mucosal resection of 10–20-mm polyps: an underappreciated problem with a simple solution? Endoscopy 57(7):750–752. 10.1055/a-2599-0604 40393658 10.1055/a-2599-0604

[40] Tabaja L, Sidani S (2023) Hybrid laparoscopic and endoscopic techniques: colon and rectum. In: Kroh M, Docimo S Jr, El Djouzi S, Shada A, Reavis KM (eds) The SAGES manual operating through the endoscope. Springer, Cham. 10.1007/978-3-031-21044-0_43

[41] van der Zander QME, van Oostendorp SEF, Bleijenberg AGC et al (2021) Endoscopic submucosal dissection versus endoscopic mucosal resection for large nonpedunculated colorectal lesions: a systematic review and meta-analysis. Gastrointest Endosc 94(5):872–886.e4. 10.1016/j.gie.2021.05.02934530974

[42] Boland P, McEntee P, Cucek J, Eržen S, Niemiec E, Petropoulou T, Burke J, Knol J, Hompes R, Tuynman J, Aigner F, Arezzo A, Cahill R (2025) Protocol for CLASSICA software as medical device trial. Minim Invasive Ther Allied Technol. 10.1080/13645706.2025.2540482 40852969 10.1080/13645706.2025.2540482

[43] Ammirati CA, Arezzo A, Gaetani C, Strazzarino GA, Faletti R, Bergamasco L, Barisone F, Fonio P, Morino M (2024) Can we apply the concept of sentinel lymph nodes in rectal cancer surgery? Minim Invasive Ther Allied Technol 33(6):334–340. 10.1080/13645706.2024.2404046 39295076 10.1080/13645706.2024.2404046

[44] Benzoni I, Fricano M, Borali J, Bonafede M, Celotti A, Tarasconi A, Ranieri V, Totaro L, Quarti LM, Dendena A, Grizzi G, Bonomi M, Grassia R, Frittoli B, Baiocchi GL (2025) Fluorescence-guided mesorectal nodes harvesting associated with local excision for early rectal cancer: technical notes. Minim Invasive Ther Allied Technol 34(4):290–296. 10.1080/13645706.2025.2473587 40042188 10.1080/13645706.2025.2473587

[45] Baldari L, Boni L, Cassinotti E (2023) Lymph node mapping with ICG near-infrared fluorescence imaging: technique and results. Minim Invasive Ther Allied Technol 32(5):213–221. 10.1080/13645706.2023.2217916 37261486 10.1080/13645706.2023.2217916

[46] Wang M (2024) Application value of SOMATOM Force computed tomography in assisting the preoperative localization of colorectal cancer resection surgery. Minim Invasive Ther Allied Technol 33(6):365–372. 10.1080/13645706.2024.2415326 39420570 10.1080/13645706.2024.2415326

