# Supplementary material for: Hybrid laparo-endoscopic techniques for challenging colorectal lesions: a systematic review
Source: Surg Endosc. 2025 Sep 29;39(11):7160–71. doi: 10.1007/s00464-025-12243-w (PMC12618300; doi:10.1007/s00464-025-12243-w)
Supplement: Supplementary file 1 — Supplementary file1 (DOCX 28 KB) [file 464_2025_12243_MOESM1_ESM.docx]

Suppl. Table 1: QUADAS criteria for potential bias

| **Potential bias** | ***Le Picard*** | ***Mal*** | ***Ommer*** | ***Winter*** | ***Wilhelm*** | ***Franklin*** | ***Grunhagen*** | ***Wood*** | ***Yan*** | ***Jang*** | ***Lee*** | ***Goh*** | ***Crawford*** | ***Lascarides*** | ***Račkauskas*** | ***Tamegai*** | ***Bulut*** | ***Suzuki*** | ***Huang*** | ***Parker*** | ***Mirza*** | ***Kolosov*** | ***Golda*** | ***Serra-Aracil*** | ***Leicher*** | ***Jones*** | ***Hanevelt,*** |
| --- | --- | --- | --- | --- | --- | --- | --- | --- | --- | --- | --- | --- | --- | --- | --- | --- | --- | --- | --- | --- | --- | --- | --- | --- | --- | --- | --- |
| 1. Was the spectrum of patients representative of the  patients who will receive the test in practice? | Y | Y | Y | Y | Y | Y | Y | Y | Y | Y | Y | Y | Y | Y | Y | Y | Y | Y | Y | Y | Y | Y | Y | Y | Y | Y | Y |
| 2. Were selection criteria clearly described? | Y | Y | N | N | Y | Y | N | Y | Y | Y | Y | Y | Y | Y | N | Y | Y | N | Y | Y | Y | N | Y | Y | Y | N | Y |
| 3. Is the reference standard likely to correctly classify  the target condition? | Y | Y | Y | Y | Y | Y | Y | Y | Y | Y | Y | Y | Y | Y | Y | Y | Y | Y | Y | Y | Y | Y | Y | Y | Y | Y | Y |
| 4. Is the time period between reference standard and  index test short enough to be reasonably sure that the  target condition did not change between the two  tests? | Y | Y | Y | Y | Y | Y | Y | Y | Y | Y | Y | Y | Y | Y | Y | Y | Y | Y | Y | Y | Y | Y | Y | Y | Y | Y | Y |
| 5. Did the whole sample or a random selection of the  sample, receive verification using a reference  standard of diagnosis? | Y | Y | Y | Y | Y | Y | Y | Y | Y | Y | Y | Y | Y | Y | Y | Y | Y | Y | Y | Y | Y | Y | Y | Y | Y | Y | Y |
| 6. Did patients receive the same reference standard  regardless of the index test result? | Y | Y | Y | Y | Y | Y | Y | Y | Y | Y | Y | Y | Y | Y | Y | Y | Y | Y | Y | Y | Y | Y | Y | Y | Y | Y | Y |
| 7. Was the reference standard independent of the  index test i. e. the index test did not form part of the  reference standard? | Y | Y | Y | Y | Y | Y | Y | Y | Y | Y | Y | Y | Y | Y | Y | Y | Y | Y | Y | Y | Y | Y | Y | Y | Y | Y | Y |
| 8. Was the execution of the index test described in  sufficient detail to permit replication of the test? | Y | N | Y | N | Y | N | N | Y | Y | Y | Y | Y | Y | Y | Y | Y | Y | N | Y | Y | Y | N | Y | Y | Y | Y | N |
| 9. Was the execution of the reference standard  described in sufficient detail to permit its replication? | Y | N | Y | N | Y | N | N | Y | Y | Y | Y | Y | Y | Y | Y | Y | Y | N | Y | Y | Y | N | Y | Y | Y | Y | N |
| 10. Were the index test results interpreted without  knowledge of the results of the reference standard? | Y | Y | Y | Y | Y | Y | Y | Y | Y | Y | Y | Y | Y | Y | Y | Y | Y | Y | Y | Y | Y | Y | Y | Y | Y | Y | Y |
| 11. Were the reference standard results interpreted  without knowledge of the results of the index test? | Y | Y | Y | Y | Y | Y | Y | Y | Y | Y | Y | Y | Y | Y | Y | Y | Y | Y | Y | Y | Y | Y | Y | Y | Y | Y | Y |
| 12. Were the same clinical data available when test  results were interpreted as would be available when  the test is used in practice? | Y | Y | Y | Y | Y | Y | Y | Y | Y | Y | Y | Y | Y | Y | Y | Y | Y | Y | Y | Y | Y | Y | Y | Y | Y | Y | Y |
| 13. Were withdrawals from the study explained? | Y | Y | Y | Y | Y | Y | Y | Y | Y | Y | Y | Y | Y | Y | Y | Y | Y | Y | Y | Y | Y | Y | Y | Y | Y | Y | Y |
