# Supplementary material for: Hybrid laparo-endoscopic techniques for challenging colorectal lesions: a systematic review
Source: Surg Endosc. 2025 Sep 29;39(11):7160–71. doi: 10.1007/s00464-025-12243-w (PMC12618300; doi:10.1007/s00464-025-12243-w)
Supplement: Supplementary file 2 — Supplementary file2 (DOCX 22 KB) [file 464_2025_12243_MOESM2_ESM.docx]

# **APPENDIX 1 - Search strategies**

**Launch Date: May 7^th^ 2025**

**PubMed**

| #1 | "Colonic Polyps"[Mesh] | 10597 |
| --- | --- | --- |
| #2 | (colorectum[tiab] OR colo-rectum[tiab] OR colorectal[tiab] OR colo-rectal[tiab] OR colon[tiab] OR colonic[tiab] OR rectum[tiab] OR rectal[tiab] OR cecum[tiab] OR caecum[tiab] OR cecal[tiab] OR caecal[tiab] OR "large intestine"[tiab] OR "large bowel"[tiab]) AND (lesion*[tiab] OR adenoma*[tiab] OR polyp*[tiab]) | 74725 |
| #3 | #1 OR #2 | 77509 |
| #4 | "surgery"[subheading] | 2459078 |
| #5 | surg*[tiab] OR resect*[tiab] OR remov*[tiab] OR excis*[tiab] OR dissect*[tiab] OR polipectom*[tiab] | 3678253 |
| #6 | #4 OR #5 | 4583236 |
| #7 | "Endoscopy"[Mesh] AND "Laparoscopy"[Mesh] | 125777 |
| #8 | endoscop*[tiab] OR colonoscop*[tiab] AND laparoscop*[tiab] | 18870 |
| #9 | endolaparoscop*[tiab] OR endo-laparoscop*[tiab] OR laparoendoscop*[tiab] OR laparo-endoscop*[tiab] | 1819 |
| #10 | #7 OR #8 OR #9 | 134277 |
| #11 | #3 AND #6 AND #10 | 2017 |
| #12 | "Clinical Trial"[Publication Type] | 1021427 |
| #13 | "Control Groups"[Mesh] OR "Double-Blind Method"[Mesh] | 185875 |
| #14 | trial[tiab] OR random*[tiab] OR group[tiab] OR groups[tiab] OR blind*[tiab] OR placebo[tiab] OR RCT[tiab] OR CCT[tiab] | 6312482 |
| #15 | NCT0*[tiab] OR NCT1*[tiab] OR NCT2*[tiab] OR NCT3*[tiab] OR NCT4*[tiab] OR NCT5*[tiab] OR NCT6*[tiab] OR NCT7*[tiab] OR NCT8*[tiab] OR NCT9*[tiab] | 134588 |
| #16 | phase-1[tiab] OR phase-I[tiab] OR phase-2[tiab] OR phase-II[tiab] OR phase-3[tiab] OR phase-III[tiab] OR phase-4[tiab] OR phase-IV[tiab] | 200043 |
| #17 | "Evaluation Study"[Publication Type] | 265935 |
| #18 | "evaluation study"[tiab] | 5152 |
| #19 | "Observational Study"[Publication Type] OR "Epidemiologic Studies"[Mesh] | 3504266 |
| #20 | observational[tiab] OR epidemiologic[tiab] OR epidemiological[tiab] OR case-control*[tiab] OR retrospective*[tiab] OR cohort*[tiab] OR longitudinal*[tiab] OR prospective*[tiab] OR cross-section*[tiab] OR follow-up[tiab] OR followup[tiab] OR "followed up"[tiab] OR transversal*[tiab] OR population-based[tiab] OR "time series"[tiab] | 4842645 |
| #21 | "Regression Analysis"[Mesh] OR "Matched-Pair Analysis"[Mesh] OR "Prevalence"[Mesh] OR "Incidence"[Mesh] | 1090636 |
| #22 | regression[tiab] OR matching[tiab] OR matched[tiab] OR prevalen*[tiab] OR incidence[tiab] | 3481584 |
| #23 | #12 OR #13 OR #14 OR #15 OR #16 OR #17 OR #18 OR #19 OR #20 OR #21 OR #22 | 11739897 |
| #24 | #11 AND #23 | 1144 |
| #25 | “Animals”[Mesh] NOT “Humans”[Mesh] | 5333385 |
| #26 | #24 NOT #25 | **1127** |

**Embase (on Embase.gov)**

| #1 | 'colon polyp'/exp | 29031 |
| --- | --- | --- |
| #2 | (colorectum:ti,ab,kw OR colo-rectum:ti,ab,kw OR colorectal:ti,ab,kw OR colo-rectal:ti,ab,kw OR colon:ti,ab,kw OR colonic:ti,ab,kw OR rectum:ti,ab,kw OR rectal:ti,ab,kw OR c$ecum:ti,ab,kw OR c$ecal:ti,ab,kw OR "large intestine":ti,ab,kw OR "large bowel":ti,ab,kw) AND (lesion*:ti,ab,kw OR adenoma*:ti,ab,kw OR polyp*:ti,ab,kw) | 124165 |
| #3 | #1 OR #2 | 135835 |
| #4 | surgery:lnk OR 'polypectomy'/exp | 2687766 |
| #5 | surg*:ti,ab,kw OR resect*:ti,ab,kw OR remov*:ti,ab,kw OR excis*:ti,ab,kw OR dissect*:ti,ab,kw OR polipectom*:ti,ab,kw | 4967983 |
| #6 | #4 OR #5 | 6212268 |
| #7 | 'endoscopy'/exp AND 'laparoscopy'/exp | 230422 |
| #8 | endoscop*:ti,ab,kw OR colonoscop*:ti,ab,kw AND laparoscop*:ti,ab,kw | 33333 |
| #9 | endolaparoscop*:ti,ab,kw OR endo-laparoscop*:ti,ab,kw OR laparoendoscop*:ti,ab,kw OR laparo-endoscop*:ti,ab,kw | 3821 |
| #10 | #7 OR #8 OR #9 | 244876 |
| #11 | #3 AND #6 AND #10 | 4958 |
| #12 | 'clinical trial'/exp | 2082174 |
| #13 | 'control group'/exp OR 'double blind procedure'/exp | 349733 |
| #14 | trial:ti,ab,kw OR random*:ti,ab,kw OR group:ti,ab,kw OR groups:ti,ab,kw OR blind*:ti,ab,kw OR placebo:ti,ab,kw OR RCT:ti,ab,kw OR CCT:ti,ab,kw | 8830346 |
| #15 | NCT0*:ti,ab,kw,cn OR NCT1*:ti,ab,kw,cn OR NCT2*:ti,ab,kw,cn OR NCT3*:ti,ab,kw,cn OR NCT4*:ti,ab,kw,cn OR NCT5*:ti,ab,kw,cn OR NCT6*:ti,ab,kw,cn OR NCT7*:ti,ab,kw,cn OR NCT8*:ti,ab,kw,cn OR NCT9*:ti,ab,kw,cn | 439635 |
| #16 | phase-1:ti,ab,kw OR phase-I:ti,ab,kw OR phase-2:ti,ab,kw OR phase-II:ti,ab,kw OR phase-3:ti,ab,kw OR phase-III:ti,ab,kw OR phase-4:ti,ab,kw OR phase-IV:ti,ab,kw | 401329 |
| #17 | 'evaluation study'/exp | 278472 |
| #18 | "evaluation study":ti,ab,kw | 6818 |
| #19 | 'observational study'/exp OR 'case control study'/exp OR 'retrospective study'/exp OR 'cohort analysis'/exp OR 'longitudinal study'/exp OR 'prospective study'/exp OR 'cross-sectional study'/exp | 4420586 |
| #20 | observational:ti,ab,kw OR epidemiologic:ti,ab,kw OR epidemiological:ti,ab,kw OR case-control*:ti,ab,kw OR retrospective*:ti,ab,kw OR cohort*:ti,ab,kw OR longitudinal*:ti,ab,kw OR prospective*:ti,ab,kw OR cross-section*:ti,ab,kw OR follow-up:ti,ab,kw OR followup:ti,ab,kw OR "followed up":ti,ab,kw OR transversal*:ti,ab,kw OR population-based:ti,ab,kw OR "time series":ti,ab,kw | 7265566 |
| #21 | 'regression model'/exp OR 'prevalence'/exp OR 'incidence'/exp | 2370986 |
| #22 | regression:ti,ab,kw OR matching:ti,ab,kw OR matched:ti,ab,kw OR prevalen*:ti,ab,kw OR incidence:ti,ab,kw | 4945234 |
| #23 | #12 OR #13 OR #14 OR #15 OR #16 OR #17 OR #18 OR #19 OR #20 OR #21 OR #22 | 16265693 |
| #24 | #11 AND #23 | 2762 |
| #25 | ('animal'/de OR 'animal experiment'/exp OR 'nonhuman'/de) NOT ('human'/de OR 'human experiment'/exp) | 7654339 |
| #26 | #24 NOT #25 | 2742 |
| #27 | #26 NOT 'conference abstract'/it | **1635** |

**Central Register of Controlled Trials (CENTRAL; 2025, issue 4)**

| #1 | MeSH descriptor: [Colonic Polyps] explode all trees | 815 |
| --- | --- | --- |
| #2 | ((colorectum OR colo-rectum OR colorectal OR colo-rectal OR colon OR colonic OR rectum OR rectal OR c?ecum OR c?ecal OR "large intestine" OR "large bowel") AND (lesion* OR adenoma* OR polyp*)):ti,ab,kw | 7450 |
| #3 | #1 OR #2 | 7450 |
| #4 | MeSH descriptor: [] explode all trees and with qualifier(s): [surgery - SU] | 88247 |
| #5 | (surg* OR resect* OR remov* OR excis* OR dissect* OR polipectom*):ti,ab,kw | 394266 |
| #6 | #4 OR #5 | 394266 |
| #7 | MeSH descriptor: [Endoscopy] this term only | 2525 |
| #8 | MeSH descriptor: [Endoscopy, Gastrointestinal] explode all trees | 6409 |
| #9 | MeSH descriptor: [Laparoscopy] explode all trees | 9244 |
| #10 | (#7 OR #8) AND #9 | 162 |
| #11 | (endoscop* OR colonoscop* AND laparoscop*):ti,ab,kw | 37095 |
| #12 | (endolaparoscop* OR endo-laparoscop* OR laparoendoscop* OR laparo-endoscop*):ti,ab,kw | 242 |
| #13 | #10 OR #11 OR #12 | 37266 |
| #14 | #3 AND #6 AND #13 | **1623** |
